# Novel paired CD13-negative (MT-50.1) and CD13-positive (MT-50.4) HTLV-1-infected T-cell lines with differential regulatory T cell-like activity

**DOI:** 10.1038/s41598-024-63494-x

**Published:** 2024-05-31

**Authors:** Yuki Egawa, Tomonori Higuchi, Yumiko Hashida, Kazuyuki Ueno, Kensuke Kojima, Masanori Daibata

**Affiliations:** 1 Department of Microbiology and Infection, Kochi Medical School, Kochi University, Nankoku, Kochi 783-8505 Japan; 2 Department of Hematology, Kochi Medical School, Kochi University, Nankoku, Kochi 783-8505 Japan; 3 Present Address: Department of Medical Laboratory Science, Faculty of Health Sciences, Kochi Gakuen University, Kochi, 780-0955 Japan

**Keywords:** Cancer, Microbiology, Oncology

## Abstract

Adult T-cell leukemia/lymphoma (ATL) occurs after human T-cell leukemia virus type-1 (HTLV-1) infection with a long latency period exceeding several decades. This implies the presence of immune evasion mechanisms for HTLV-1-infected T cells. Although ATL cells have a CD4^+^CD25^+^ phenotype similar to that of regulatory T cells (Tregs), they do not always possess the immunosuppressive functions of Tregs. Factors that impart effective immunosuppressive functions to HTLV-1-infected cells may exist. A previous study identified a new CD13^+^ Treg subpopulation with enhanced immunosuppressive activity. We, herein, describe the paired CD13^−^ (designated as MT-50.1) and CD13^+^ (MT-50.4) HTLV-1-infected T-cell lines with Treg-like phenotype, derived from the peripheral blood of a single patient with lymphoma-type ATL. The cell lines were found to be derived from HTLV-1-infected non-leukemic cells. MT-50.4 cells secreted higher levels of immunosuppressive cytokines, IL-10 and TGF-β, expressed higher levels of Foxp3, and showed stronger suppression of CD4^+^CD25^−^ T cell proliferation than MT-50.1 cells. Furthermore, the CD13 inhibitor bestatin significantly attenuated MT-50.4 cell growth, while it did not for MT-50.1 cells. These findings suggest that CD13 expression may be involved in the increased Treg-like activity of MT-50.4 cells. Hence, MT-50.4 cells will be useful for in-depth studies of CD13^+^Foxp3^+^ HTLV-1-infected cells.

## Introduction

Adult T-cell leukemia/lymphoma (ATL) is an aggressive mature T-cell malignancy caused by human T-cell leukemia virus type-1 (HTLV-1). ATL occurs in approximately 5% of individuals infected with HTLV-1 and has a long latency period exceeding several decades^1–3^. How HTLV-1-infected cells can survive for such a long time necessary for ATL development remains unclear. Immune suppression associated with HTLV-1 infection likely contributes to the progression to clinically apparent ATL^3,4^. ATL cells have a CD4^+^CD25^high^ phenotype similar to that of regulatory T cells (Tregs). Foxp3, which is a Treg-cell-specific transcription factor, is expressed in HTLV-1-infected cells in a subset of patients with ATL^5–7^. Its expression is critical for the function of Tregs, including peripheral tolerance and inhibition of effector T cells^8,9^. Although some studies have described an immunosuppressive effect of ATL cells on the proliferation of conventional T cells^10,11^, Foxp3^+^ ATL cells and Foxp3^+^ HTLV-1-infected non-leukemic cells do not always possess the immunosuppressive functions of Tregs^12–14^. In this context, identifying the factors that impart more effective immunosuppressive functions to HTLV-1-infected cells can help understand the mechanisms by which HTLV-1 causes immune evasion and the severe immunocompromised state observed in some patients with ATL.

CD13 is not only recognized as a good myeloid lineage marker but also as an aminopeptidase N^15^. A previous study identified a new Treg subpopulation, CD13-expressing CD4^+^CD25^high^ Tregs, among CD4^+^CD25^high^ Tregs in the peripheral blood of patients with non-small cell lung cancer and found that CD13^+^ Tregs with high levels of Foxp3 expression exhibited enhanced immunosuppressive functions^16^. Therefore, it can be hypothesized that if CD13 is also expressed in CD4^+^CD25^high^ HTLV-1-infected cells in patients with ATL or individuals carrying HTLV-1, those cells may have increased immunosuppressive functions. However, difficulties in isolating a large number of such cells are major obstacles to testing this hypothesis. The availability of cell lines will likely facilitate a better understanding of the function of the unique type of CD13^+^ Treg-like cells and aid the discovery of pharmacological reagents to suppress these cells because cell lines provide unlimited supplies of cells using which studies can be performed repeatedly and extensively.

We established two CD13^−^ (designated as MT-50.1) and CD13^+^ (MT-50.4) HTLV-1-infected cell lines with Treg-like phenotype derived from the peripheral blood of a single patient with lymphoma-type ATL. Comparison of the paired cell lines with the same origin provided an opportunity to study the biological role of CD13^+^CD4^+^CD25^+^ HTLV-1-infected cells. Using these cell lines, we showed that the CD13^+^ HTLV-1-infected Treg-like cells exhibited increased immunosuppressive functions compared with the CD13^−^ HTLV-1-infected Treg-like cells.

## Methods

### Case history and cell culture

The MT-50.1 and MT-50.4 cell lines were established from the peripheral blood of a 68-year-old Japanese woman with lymphoma-type ATL. Pathology of the cervical lymph node biopsy specimen showed atypical tumor cells with the phenotype of CD3^+^, CD4^+^, CD8^−^, CD13^−^, CD20^−^, and CD25^+^ (Supplementary Fig. S1a). The online supplement provides the details of the procedures of immunohistochemistry. The antibodies used are listed in Supplementary Table S1. Southern blot analysis demonstrated monoclonal integration of the HTLV-1 provirus genome in the tumor cells (Supplementary Fig. S1b). Giemsa-banding chromosomal analysis of the tumor cells showed a complex karyotype (Supplementary Fig. S1c). The patient was treated with four courses of combination chemotherapy comprising cyclophosphamide, doxorubicin, ranimustine, vindesine, etoposide, carboplatin, and prednisolone (modified VCAP-AMP-VECP). Remission had been maintained for more than 4 years.

The white blood cell count at diagnosis was 7.5 × 10^9^/L with 63% neutrophils, 1% basophils, 3% monocytes, 8% lymphocytes, and 25% atypical cells with folded or cleaved nuclei. The peripheral blood mononuclear cells (PBMCs) were separated using Ficoll-Paque PLUS (Cytiva, Tokyo, Japan) gradient centrifugation. The cells were seeded separately in four cell culture dishes and cultured in RPMI 1640 medium supplemented with 20% heat-inactivated fetal bovine serum (FBS) and 100 U/mL recombinant human interleukin-2 (IL-2, Shionogi Pharma, Osaka, Japan). The cultures were incubated at 37 °C with 5% CO_2_ in the air, and their media were partially changed twice a week.

The presence of the Epstein–Barr virus (EBV) genomes was examined using polymerase chain reaction (PCR) with specific primers for the *Bam*HI-W (TC60/TC61) fragments^17^.

Among the ATL-derived T-cell lines used in this study, ST1 and KOB depend on IL-2 for their growth, whereas MT-1, ATL-1 K, and H582 are IL-2 independent^18–20^. MT-2 and SLB-1 are HTLV-1-infected T-cell lines derived from normal T cells^2,21^. SezM3 is an IL-2-dependent HTLV-1-infected non-leukemic T-cell line from an HTLV-1 carrier^22^. Jurkat is an HTLV-1-negative T-cell line^23^.

This study was approved by the Ethics Committee of Kochi Medical School, Kochi University, Japan (approval no. 22-24). Written informed consent was provided by the patient and donors, and all experiments were performed following the regulations of the institutional review board.

### HTLV-1 clonality assay

HTLV-1 provirus integration profile was examined using southern blot analysis. High molecular weight DNA was digested using *Eco*RI, which does not cut typical provirus DNA, or *Pst*I, which cuts it at multiple sites. The digests were separated using agarose gel electrophoresis. The DNAs in the gel were blotted onto a nitrocellulose filter and hybridized with an HTLV-1 probe^24^. Non-leukemic cell line was defined as one that did not have the same southern blotting pattern as the patient’s leukemic cells^22^.

### HTLV-1 mRNA expression

Real-time quantitative reverse-transcription PCR (qRT–PCR) was used to measure the expression levels of HTLV-1 *tax*, and *HBZ* mRNAs. Total RNA was extracted and purified using TRIzol reagent (Thermo Fisher Scientific, Tokyo, Japan) and Direct-zol DNA/RNA Miniprep kits (Zymo Research, Irvine, CA, USA). The total RNA was treated with DNase to avoid any amplification of genomic DNA and was reverse-transcribed using SuperScript IV VILO master mix (Thermo Fisher Scientific). An aliquot of each cDNA was subjected to qPCR analysis. The reaction was conducted in triplicate on a StepOnePlus thermocycler (Thermo Fisher Scientific) with KOD SYBR qPCR mix (Toyobo, Osaka, Japan) containing 0.4 μM of each primer. The primer sequences used to determine the gene expression are listed in Supplementary Table S2. The PCR conditions were 2 min at 98 °C, followed by 45 cycles of 10 s at 98 °C, 10 s at 63 °C, and 30 s at 68 °C. Relative gene expression levels were calculated using 2^−ΔCt^ values, with the *β-actin* gene (*ACTB*) as a housekeeping control.

### Chromosome analysis

Metaphase chromosome spreads were G-banded according to standard procedures^25^. Karyotypes have been described according to the International System for Cytogenetic Nomenclature.

### Short tandem repeat (STR) DNA fingerprinting

STR DNA fingerprinting was performed using a GenePrint 10 System (Promega, Madison, WI, USA), which allowed coamplification and detection of 10 human loci, namely, *TH01*, *D21S11*, *D5S818*, *D13S317*, *D7S820*, *D16S539*, *CSF1PO*, *Amelogenin*, *vWA*, and *TPOX*. To determine the similarity between STR profiles from two different cells, we used an evaluation value (EV). They were considered identical when the EV value was 0.9 or higher^26^.

### Flow cytometry

Cells were first stained using a Zombie Aqua Fixable Viability kit (BioLegend, San Diego, CA, USA) to differentiate between live and dead cell populations. For staining of cell surface markers, cells were incubated with fluorescently labeled antibodies or isotype-matched controls. For Foxp3 staining, cells were fixed and permeabilized using Fixation/Permeabilization Concentrate and Diluent (Thermo Fisher Scientific) and stained using a fluorescently labeled anti-human Foxp3 antibody. The antibodies used are listed in Supplementary Table S3. The cells were analyzed using a flow cytometer and FlowJo software (Tree Star Inc., Ashland, OR, USA).

### Enzyme-linked immunosorbent assay (ELISA)

Cells were seeded in a 24-well plate at a density of 4 × 10^5^/mL and cultured for 3 days. Concentrations of IL-10 and transforming growth factor (TGF)-β in the culture supernatants were measured using DuoSet ELISA kits (R&D Systems, Minneapolis, MN, USA).

### Cell isolation and in vitro suppression assay

CD4^+^ T cells were isolated from PBMCs of two healthy donors, a 47-year-old male (donor 1) and a 23-year-old male (donor 2), using a MojoSort Human CD4 Naïve T Cell Isolation kit (BioLegend). Subsequently, CD4^+^CD25^−^ conventional T cells and CD4^+^CD25^+^CD127^−^ Tregs were isolated from the CD4^+^ T cell population using MojoSort streptavidin nanobeads (BioLegend). Conventional CD4^+^CD25^−^ T cells were negatively selected from CD4^+^ T cells incubated with biotin-labeled antibodies against human CD25 (Biolegend). Treg cells were negatively selected from CD4^+^ T cells incubated with biotin-labeled antibodies against human CD127 (Biolegend), followed by positive selection using biotin-labeled anti-human CD25 antibody. For Treg expansion, the purified Tregs were cultured with 500 U/mL of human IL-2 and Dynabeads Human T-Activator CD3/CD28 beads (Thermo Fisher Scientific) for 7 days. To prepare dendritic cells, CD14^+^ monocytes were isolated from PBMCs using a MojoSort Human CD14^+^ Monocytes Isolation kit (BioLegend), and the purified monocytes were differentiated into mature dendritic cells using an ImmunoCult Dendritic Cell Culture kit (Stemcell Technologies, Vancouver, Canada).

MT-50.1 or MT-50.4 cells (suppressor cells [SCs]) were evaluated for suppressor activity in coculture assay with the healthy donor-derived CD4^+^CD25^−^ cells (responder cells [RCs]). RCs were labeled using 5 μM of carboxyfluorescein succinimidyl ester (CFSE, BioLegend) and then incubated in wells of 96-well plates (5 × 10^4^/well) containing Cell Therapy Systems AIM-V serum-free medium (Thermo Fisher Scientific) and human IL-2 (100 U/mL) at RC:SC ratios of 2:1, 4:1, and 8:1. RCs were stimulated using 10 μg/mL of anti-human CD3 antibody (clone OKT3, BioLegend) in the presence of mature dendritic cells (5 × 10^3^/well). On day 5 of culture, the cell proliferation of CFSE-labeled RCs was analyzed using flow cytometry.

### Cell proliferation, apoptosis, and cell cycle analyses

Cells were seeded into 96-well plates (2 × 10^4^/well) and treated with bestatin (Selleck Chemicals, Houston, TX, USA) at concentrations ranging from 0.2 μg/mL to 50.0 μg/mL. For cell proliferation assays, viable cells were counted every 24 h using a flow cytometer by gating out cells stained with propidium iodide. For cell cycle analysis, cells were fixed in cold 70% ethanol, treated with RNase, and stained with propidium iodide on day 3 after the bestatin treatment. For apoptosis analysis, cells were stained with annexin V-phycoerythrin and 7-amino-actinomycin D on day 3 after the bestatin treatment. All flow cytometry data were analyzed using an Attune Acoustic Focusing Cytometer (Thermo Fisher Scientific) and FlowJo software.

### Statistical analysis

The normal distribution of data was assessed using the Shapiro–Wilk test. Differences between pairs of groups were analyzed using the Mann–Whitney nonparametric *U* test. Differences between multiple groups were analyzed using a one-way analysis of variance with the Tukey post hoc test. Significance was set at a *P*-value < 0.05.

## Results

### Establishment of the MT-50.1 and MT-50.4 cell lines

**Figure 1:**
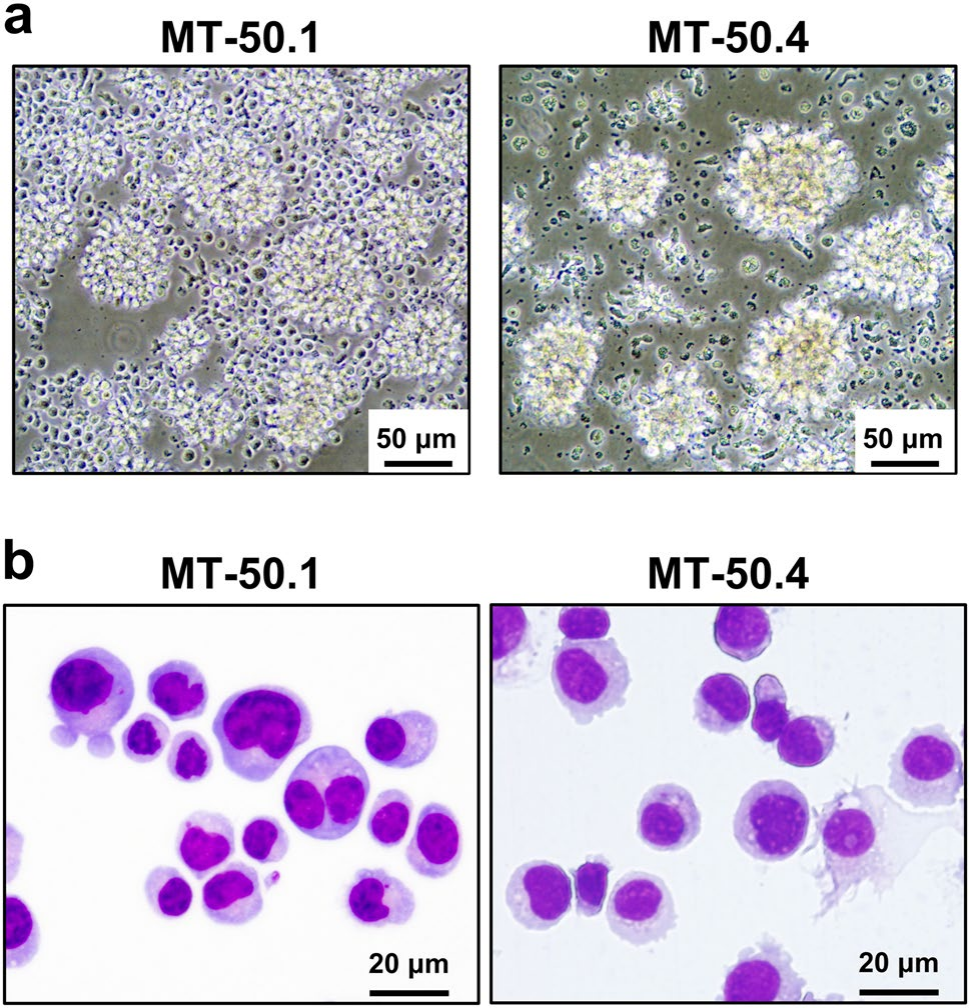
Morphology of MT-50.1 and MT-50.4 cells. (**a**) Phase-contrast microphotograph of growing cells. (**b**) Cytospin preparation of cells (May–Giemsa staining).

The cells started to proliferate 3 months after the initiation of culture in two of the four culture dishes, and then they could be regularly passaged once a week in RPMI 1640 medium supplemented with 10% FBS. The growth of both cell lines was dependent on IL-2. After 20 passages, they were considered continuous cell lines and named MT-50.1 and MT-50.4. These two cell lines could be frozen in CELLBANKER (Takara Bio, Shiga, Japan) and revived after liquid nitrogen storage. MT-50.1 and MT-50.4 cells grew as single-cell suspensions containing many cell aggregates (Fig. 1a), with a doubling time of 18 h and 24 h, respectively. Morphologically, the two cell lines were similar (the nuclei were round or slightly irregular with slightly coarse chromatin), but folded or cleaved nuclei characteristic of ATL cells were not prominent (Fig. 1b). These cell lines were found to be negative for EBV using PCR analysis. They were passaged for more than 200 passages with weekly medium changes. However, they lost viability in the absence of IL-2. The cell viability of MT-50.1 and MT-50.4 was reduced by approximately 30% and 60%, respectively, after 3 days of culture without IL-2 (Supplementary Fig. S2).

### Cell origin of MT-50.1 and MT-50.4 cell lines

**Figure 2:**
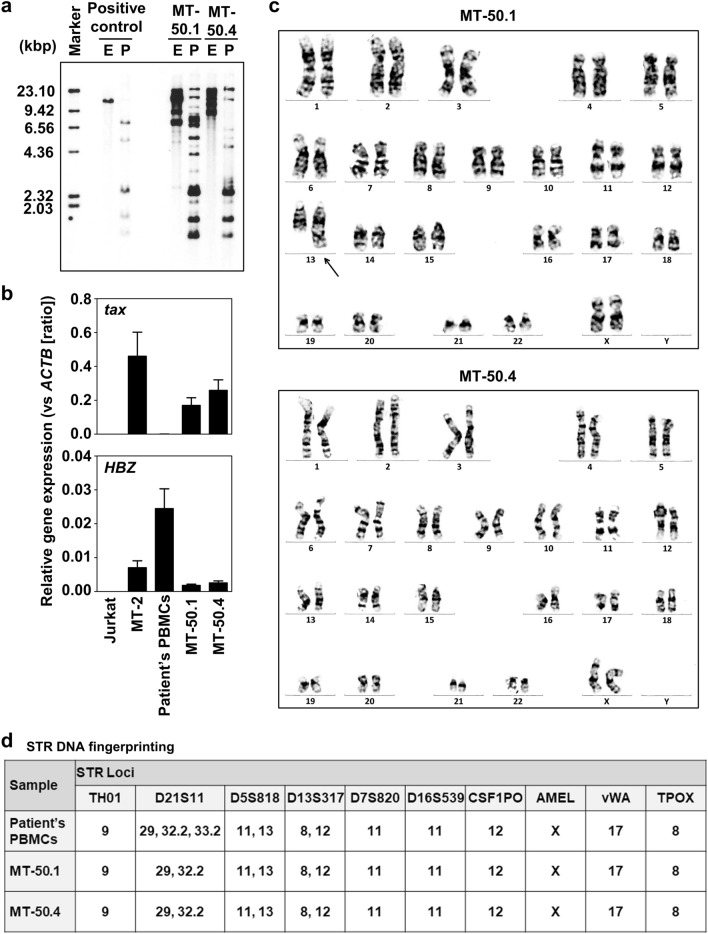
Viral and genetic analyses of MT-50.1 and MT-50.4 cells. (**a**) HTLV-1 clonality assay in MT-50.1 and MT-50.4 cells, showing multiple integrations of the HTLV-1 provirus genome. E, *Eco*RI; P, *Pst*I. The original blot photograph is presented in Supplementary Figure S7. (**b**) Expression analysis of the HTLV-1 *tax* and *HBZ* genes using qRT–PCR. Relative mRNA expression levels were calculated using 2^−ΔCt^ values, with the β-actin gene (*ACTB*) used as a housekeeping control. Results from three separate experiments are shown. Jurkat, which is an HTLV-1-negative T-cell line, was included as a negative control. MT-2, which is an HTLV-1-infected T-cell line derived from normal T cells, was included as a positive control for the viral gene expression. (**c**) Giemsa-banded karyotype, showing the following karyotype: MT-50.1, 46, XX, der(13)t(2;13)(q11.2;q34); MT-50.4, 46, XX. All 20 analyzed metaphases showed the same karyotype in each cell line. Arrows indicate structural chromosomal abnormalities. (**d**) STR profiles of the patient’s PBMCs and the established cell lines demonstrating that MT-50.1 and MT-50.4 cells were derived from the patient’s cells.

HTLV-1 clonality assay showed that these cell lines had multiple clones of HTLV-1 provirus with different genomic integration sites (Fig. 2a). The viral integration pattern differed from that of the primary ATL cells with monoclonal viral integration. These findings suggested that MT-50.1 and MT-50.4 cell lines were derived from different HTLV-1-infected non-leukemic cells that existed in the patient’s peripheral blood. We next examined the expression of the HTLV-1 *tax* and *HBZ* genes using qRT-PCR (Fig. 2b). Previous studies showed that *tax* was silenced or downregulated in the majority of ATL cells in vivo and ATL cell lines, while *HBZ* was expressed in both leukemic and non-leukemic HTLV-1-infected cells^22^. Consistent with the findings of HTLV-1-infected non-leukemic cell lines, MT-50.1 and MT-50.4 cells expressed both *tax* and *HBZ*. Chromosome analysis performed after 20 passages of the cell lines showed the different karyotypes (a normal karyotype in MT-50.4 cells and a more complicated karyotype in MT-50.1 cells, Fig. 2c). The karyotypes of the two cell lines also differed from that of the primary ATL cells. We next performed STR identification to confirm that the cell lines originated from the patient’s cells (Fig. 2d and Supplementary Fig. S3). STR profile based on the genotyping of 10 loci in the patient’s PBMCs was consistent with those of MT-50.1 and MT-50.4 cells as determined using the EV^26^, which was 1.00 between MT-50.1 and MT-50.4, and 0.96 between the patient’s cells and MT-50.1/MT-50.4. These findings demonstrate that the cell lines were indeed derived from the patient’s cells. Thus, we considered that MT-50.1 and MT-50.4 were the paired IL-2-dependent cell lines derived from the different HTLV-1-infected non-leukemic cells of a single patient with ATL.

### CD13 expression in MT-50.4 cell line

Flow cytometric analysis showed that MT-50.1 and MT-50.4 cells were positive for CD2, CD3, CD4, CD5, CD25, and CD30, while negative for CD7, CD8, CD10, CD19, CD20, CD22, CD23, CD34, CD38, CD41, CD56, CD79a, and CD103 (Supplementary Fig. S4 and Supplementary Table S4). Both cell lines did not express myelomonocyte markers, including myeloperoxidase, CD11c, CD14, and CD16, but MT-50.4 cells were strongly positive for CD13. Thus, the immunophenotype profiles of MT-50.1 and MT-50.4 were similar, except for CD13 expression in MT-50.4. The CD13 positivity in MT-50.4 cells was maintained even after 200 passages.

**Figure 3:**
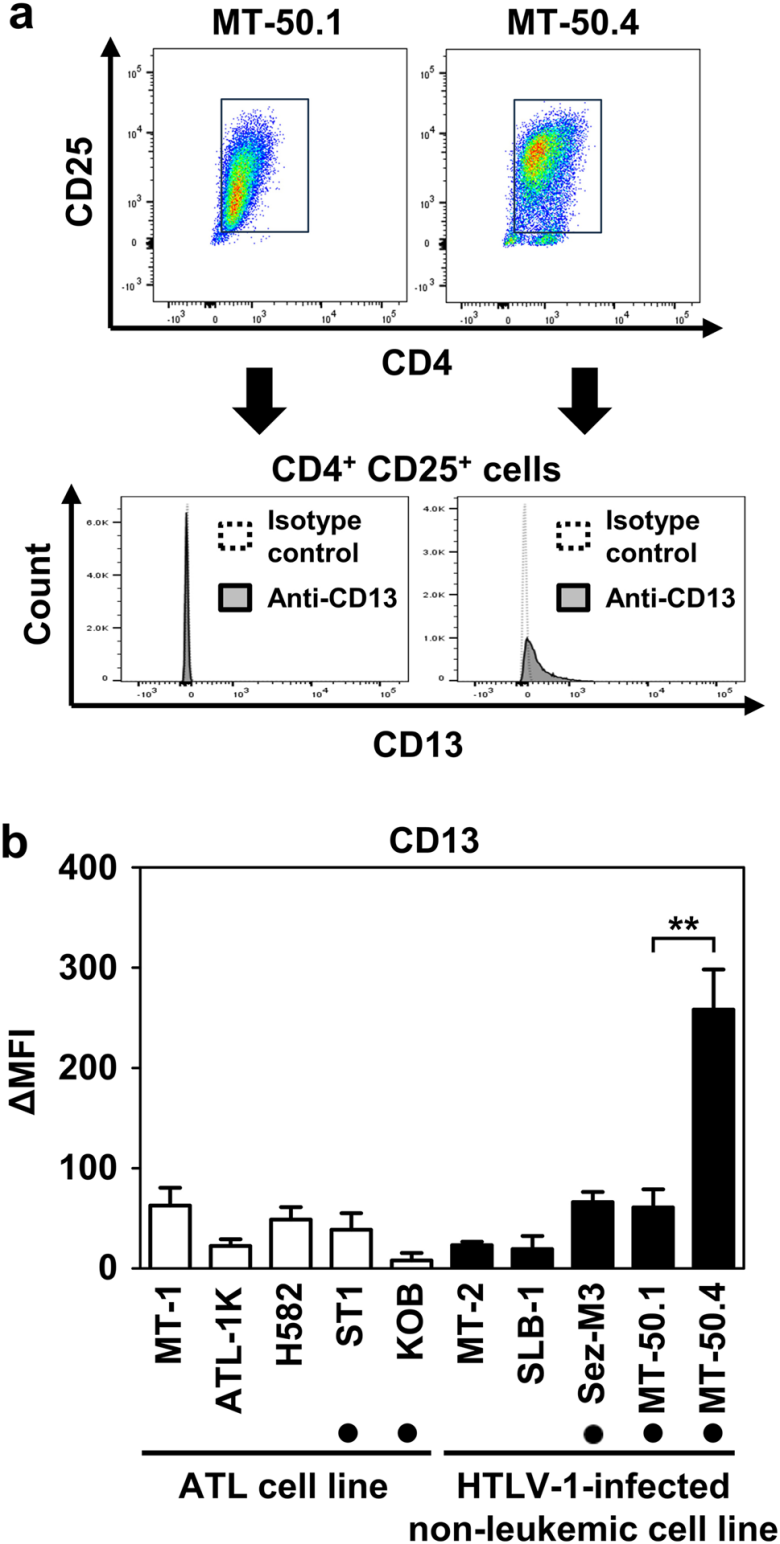
CD13 expression analysis in MT-50.1 and MT-50.4 cells. (**a**) CD13 expression in CD4^+^CD25^+^ cells. CD4^+^CD25^+^ cells were isolated using flow cytometric sorting and analyzed for CD13 expression. (**b**) CD13 expression in CD4^+^CD25^+^ cells from various ATL cell lines and HTLV-1-infected non-leukemic cell lines. CD13 expression level was represented as delta median fluorescence intensity (ΔMFI), calculated as the MFI of CD13 staining minus the MFI of the isotype control. The IL-2-dependent cell line is indicated using a filled circle. Data are shown as the mean ± standard error of the mean (SEM) of three independent experiments. Significant differences between MT-50.1 and MT-50.4 cells are shown as **P < 0.01.

In further analysis, we gated the CD4^+^CD25^+^ cell population using flow cytometric cell sorting and confirmed the CD13 expression in MT-50.4 (Fig. 3a). Although the percentage of the CD4^+^CD25^+^ cells in MT-50.1 and MT-50.4 cells was over 90%, the median fluorescent intensities (MFI) of CD25 in those cell populations were 2026 ± 187 and 5387 ± 871, respectively. These findings suggested that MT-50.4 cells expressed higher levels of CD25 than MT-50.1 cells. When comparing the MFI of CD13, the CD4^+^CD25^+^ cells in the MT-50.4 cell population expressed significantly higher levels of CD13 than those in any other HTLV-1-infected cell lines tested, regardless of whether they were IL-2 dependent or independent for cell growth, or whether they were leukemic or non-leukemic cells (Fig. 3b).

### Higher levels of secretion of IL-10 and TGF-β by MT-50.4 cell line

**Figure 4:**
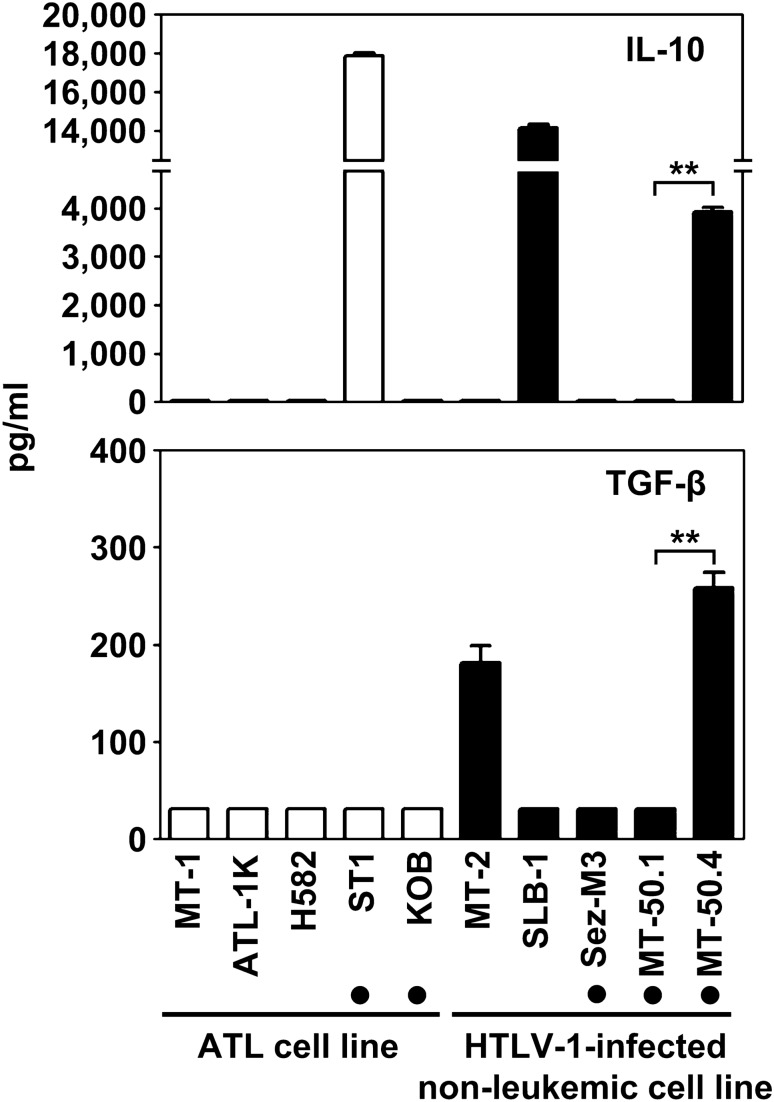
Secretion of IL-10 and TGF-β by MT-50.1 and MT-50.4 cells. ATL cell lines and other HTLV-1-infected non-leukemic cell lines were also included in this study. A total of 4 × 10^5^/mL cells were seeded into 24-well plates and cultured for 3 days. Concentrations of IL-10 and TGF-β in the cell-free culture supernatants were measured using ELISA. The TGF-β concentrations were below the detection limit (31.3 pg/mL) in culture supernatants of MT-50.1 cells. The IL-2-dependent cell line is indicated using a filled circle. Data are shown as the mean ± SEM of three independent experiments. Significant differences between MT-50.1 and MT-50.4 cells are shown as **P < 0.01.

We next examined the secretion of the immunosuppressive cytokines, IL-10 and TGF-β, by MT-50.1 and MT-50.4 cells in the culture medium. Although some HTLV-1-infected cell lines produced either IL-10 or TGF-β, MT-50.4 cells secreted both IL-10 and TGF-β at relatively high levels (Fig. 4). By contrast, the cytokine concentrations in culture supernatants of MT-50.1 cells were below the detection limits in our ELISA assays. These findings indicate that MT-50.4 cells can produce high levels of immunosuppressive cytokines. Therefore, we assumed that MT-50.4 cells may have a stronger suppressive activity similar to that of Tregs than MT-50.1 cells.

### High levels of CD13 expression in Treg-like cells of MT-50.4 cell line

**Figure 5:**
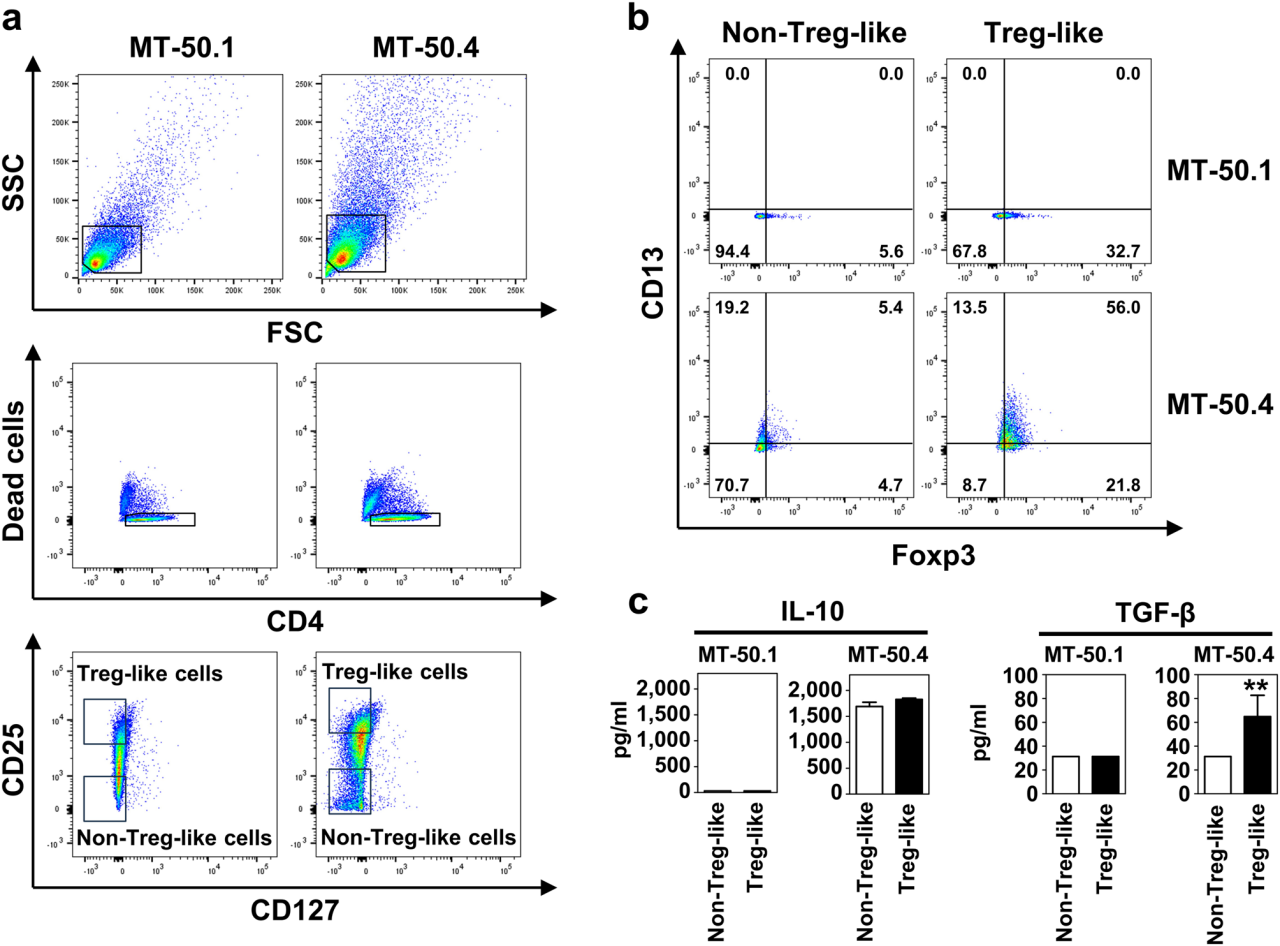
CD13 expression in Treg-like cells in MT-50.1 and MT-50.4 cell populations. (**a**) Gating strategy of flow cytometry for sorting Treg-like cell population. We gated the cells using forward scatter (FSC) and side scatter (SCC), followed by the exclusion of dead cells, and then identified CD4^+^ cells. Among these CD4^+^ cells, we gated the CD25^high^CD127^−^and the CD25^low^CD127^−^ cell subpopulations. (**b**) Flow cytometric analysis for expression of Foxp3 and CD13 in the CD4^+^CD25^high^CD127^−^ Treg-like cells and the CD4^+^CD25^low^CD127^−^ non-Treg-like cells. Foxp3^+^ cells in the MT-50.4 cell population expressed high levels of CD13. (**c**) Measurement of secreted IL-10 and TGF-β concentrations by ELISA in the cell-free culture supernatant of the Treg-like and non-Treg-like cell subpopulations. A total of 4 × 10^5^/mL cells were cultured for 3 days. The TGF-β concentrations were below the detection limit (31.3 pg/mL) in culture supernatants of MT-50.1 cells. Data are shown as the mean ± SEM of three independent experiments. Significant differences are shown as **P < 0.01.

The absence of CD127 on CD4^+^ cells can be used as a marker of Tregs^27^. We isolated the CD4^+^CD25^high^CD127^−^ Treg-like cell subpopulation and the CD4^+^CD25^low^CD127^−^ non-Treg-like cell subpopulation using flow cytometry and examined the expression of Foxp3 and CD13 in each cell population (Fig. 5a, b). In MT-50.4 cells, more than 70% of the Foxp3^+^CD4^+^CD25^high^CD127^−^ cells expressed CD13, whereas only 20% of the Foxp3^−^CD4^+^CD25^low^CD127^−^ cells were positive for CD13. Thus, MT-50.4 cells harbored a rich population of Foxp3^+^CD4^+^CD25^high^CD127^−^ Treg-like cells that expressed a high level of CD13, while the Foxp3^+^CD4^+^CD25^high^CD127^−^ Treg-like cells in the MT-50.1 cell population did not express CD13. We also found that the Treg-like cells in the MT-50.4 cell population secreted significantly higher levels of TGF-β than non-Treg-like cells (Fig. 5c). However, the secretion levels of IL-10 were not significantly different.

### Higher suppressive activity of MT-50.4 cell line

**Figure 6:**
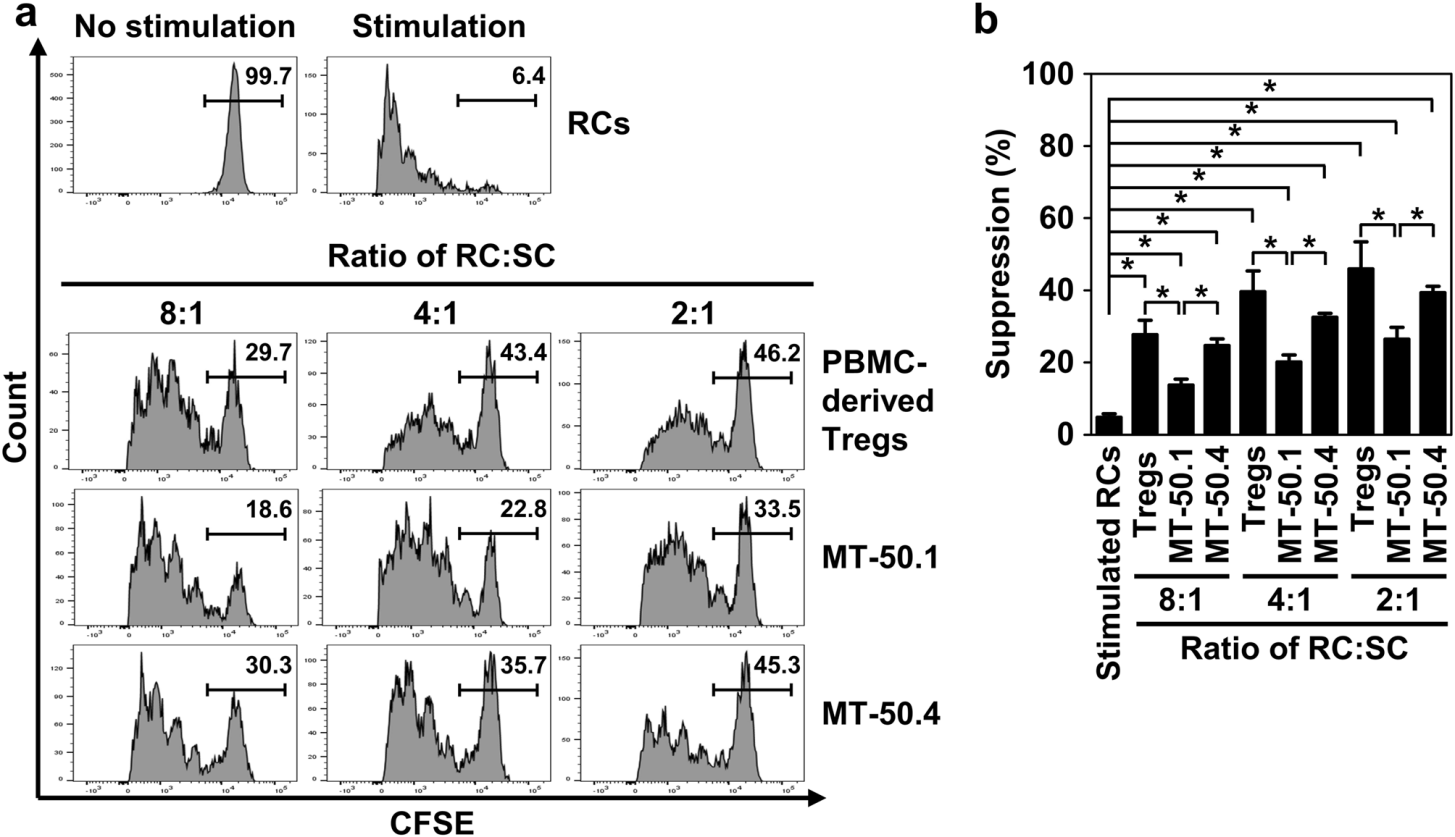
Suppressive activity of MT-50.1 and MT-50.4 cells. (**a**) Flow cytometry histogram showing the increased immunosuppressive function of MT-50.4 cells. CFSE-labeled CD4^+^CD25^−^ T cells (5 × 10^4^, responder cells [RCs]) were cocultured with suppressor cells (SCs; PBMC-derived Tregs obtained from a healthy donor [positive suppressor control], MT-50.1, or MT-50.4 cells), in the presence of mature dendric cells (5 × 10^3^), 100 U/mL of human IL-2, and 10 μg/mL of the anti-human CD3 monoclonal antibody OKT3. The SCs were serially diluted, keeping the same number of RCs at RC:SC ratios of 2:1, 4:1, and 8:1. After 5 days of culture, the CFSE intensity of RCs was measured using a flow cytometer. The dividing cells are toward the left side in the flow cytometry histogram. The proportion (%) of cells that failed to undergo cell division is indicated in the figure. Unstimulated and stimulated RCs in the absence of SCs were used as negative and positive controls for cell division, respectively. (**b**) Bar graph showing percent suppression of cell division. Data are shown as the mean ± SEM of three independent experiments. Significant differences are shown as *P < 0.05.

Tregs can functionally suppress activated T-cell proliferation. Therefore, we evaluated the suppressive effect of MT-50.1 and MT-50.4 cells (defined as SCs) on the proliferation of CD4^+^CD25^−^ T cells (defined as RCs). We cocultured MT-50.1 or MT-50.4 cells with CFSE-labeled stimulated RCs isolated from PBMCs of healthy donors. PBMC-derived Tregs isolated from the donors, which were used as positive suppressor controls, were also cocultured with the autologous RCs. Representative data using RCs from donor 1 are shown in Fig. 6a. Similar to the suppressive activity of PBMC-derived Tregs, both MT-50.1 and MT-50.4 cells inhibited the proliferation of the stimulated RCs in an RC:SC ratio-dependent manner. Notably, the suppression levels induced by MT-50.4 cells were significantly higher than those by MT-50.1 cells (Fig. 6b). The independent suppression assay using RCs obtained from donor 2 also showed that MT-50.4 cells suppressed the RC proliferation significantly more than MT-50.1 cells (Supplementary Fig. S5). These findings suggest that MT-50.4 cells have a higher ability to suppress the proliferation of conventional CD4^+^CD25^−^ T cells than MT-50.1 cells.

### Antiproliferative effect of bestatin

**Figure 7:**
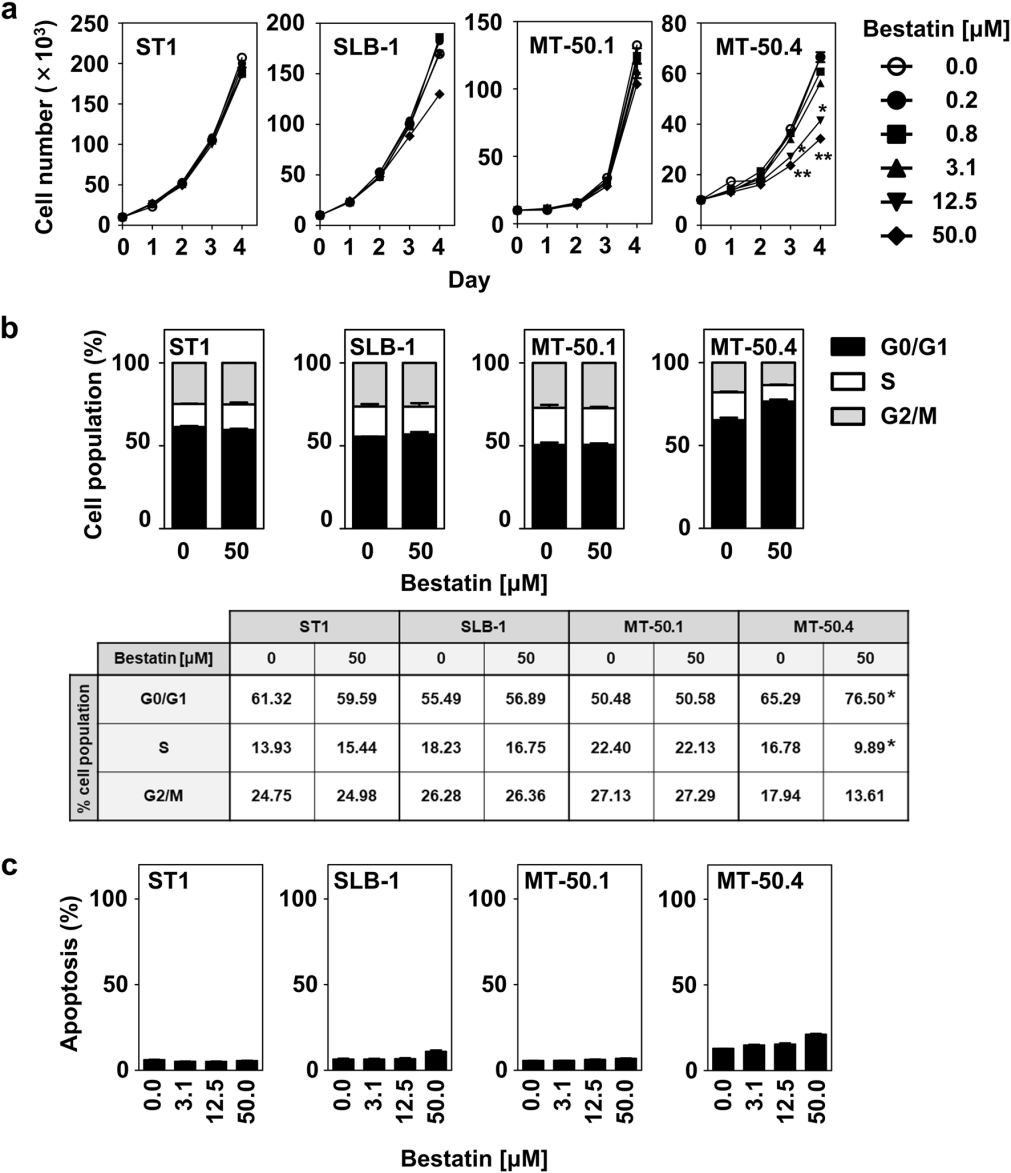
Effects of bestatin on cell proliferation, cell cycle, and apoptosis in MT-50.1 and MT-50.4 cells. (**a**) Cell proliferation assay. Cells were cultured in the presence of various concentrations of bestatin, and viable cells were counted every 24 h. After 3 days of culture, exposure of the cells to bestatin reduced the viable cell number in the case of MT-50.4 cells, while MT-50.1 and the CD13^−^ HTLV-infected cell lines ST1 and SLB-1 were not sensitive to bestatin. (**b**) Cell cycle analysis. Cells were treated with bestatin at a concentration of 50 μM for 3 days, and cell cycle stage distributions were determined. Percentages of the cell population in each stage of the cell cycle are presented outside the graph. (**c**) Apoptosis assay. Cells were treated with bestatin at the indicated concentrations for 3 days. The graphs show the percentage of apoptotic cells in the total cell population. All experiments were independently repeated three times, and data are expressed as the mean ± SEM. Significant expression differences are shown as *P* < 0.05; **P < 0.01.

Previous studies showed that most CD13-positive myeloblastic and monocytic leukemia cell lines were sensitive to bestatin, which is an inhibitor of CD13^28^. Therefore, we evaluated the growth inhibitory activity of bestatin in MT-50.1 and MT-50.4 cells. The HTLV-1-infected ST1 and SLB-1 cell lines were also included in this study. The cells were incubated with various concentrations of bestatin for 4 days. The treatment significantly attenuated cell growth of MT-50.4 cells in a dose-responsive manner (Fig. 7a), while it did not induce significant suppression in ST1, SLB-1, or MT-50.1 cells. The estimated half-maximal inhibitory concentration value of bestatin for MT-50.4 cells on day 4 of incubation was 45 μM. We next conducted experiments to determine whether the antiproliferative effects would be mediated through cell cycle inhibition and/or apoptosis. Bestatin caused a significant increase in the proportion of cells in the G0/G1 phase in the MT-50.4 cell population, while it had a limited effect on apoptosis (Fig. 7b, c). The treatment did not induce significant cell cycle arrest or apoptosis in ST1, SLB-1, or MT-50.1 cells, even at a high concentration of 50 μM.

## Discussion

We generated and characterized two phenotypically distinct HTLV-1-infected T-cell lines, MT-50.1 and MT-50.4, derived from the peripheral blood of a single patient with lymphoma-type ATL. MT-50.4 cells expressed a high level of CD13 but not MT-50.1 cells. The features of these cell lines are summarized in Table 1. The derivation of these cell lines was authenticated using STR profiling.

**Table 1:** Features of the MT-50.1 and MT-50.4 cell lines.

Parameter	Features	
	MT-50.1	MT-50.4
Clinical data		
Patient	68-year-old woman	
Diagnosis	Lymphoma-type ATL	
Sample	Peripheral blood	
Sample collection time	At diagnosis	
Year of establishment	2018	
Cell line		
Authentication	Yes (by STR DNA fingerprinting)	
Culture medium	RPMI 1640 supplemented with FBS (10%) and human IL-2 (100 U/mL)	
Growth pattern	Single-cell suspension with cell aggregates	
Cryopreservation	CELLBANKER (Takara Bio)	
Morphology	Medium-to-large-sized cells with round or slightly irregular nuclei	
Viral status	Polyclonal integration of the HTLV-1 genome	
Expression of HTLV-1 *tax* and *HBZ*	+	
Doubling time	18 h	24 h
Karyotype	46, XX, der(13)t(2;13)(q11.2;q34)	46, XX
Immunoprofile	CD4^+^CD13^−^CD25^+^	CD4^+^CD13^+^CD25^+^
Secretion of IL-10	−	+
Secretion of TGF-β	Below the detection limit^a^	+
Suppression effect on CD4^+^ T cells	Lower^b^	Higher^b^
Efficacy of bestatin on cell growth inhibition	−	+

ATL, adult T-cell leukemia/lymphoma; FBS, fetal bovine serum; HTLV-1, human T-cell leukemia virus type-1; IL-2, interleukin-2; STR, short tandem repeat; TGF-β, transforming growth factor-β.

^a^ELISA assay using human TGF-β DuoSet ELISA kit.

^b^Comparison between the MT-50.1 and MT-50.4 cells.

Regarding the origin of MT-50.1 and MT-50.4 cells, no clonal continuity was observed between the primary ATL cells and the established cell lines. Based on the findings of the chromosome analysis and the HTLV-1 clonality assay, we considered that MT-50.1 and MT-50.4 cells were derived from different HTLV-1-infected non-leukemic cells. To exclude the possibility that the IL-2 addition might have induced CD13 expression in MT-50.4 cells, while it did not induce CD13 expression in MT-50.1 cells, we examined whether IL-2 stimulation could lead to *CD13* mRNA expression in CD13^−^ HTLV-1-infected cells using qRT-PCR (Supplementary Fig. S6). We did not observe *CD13* expression up to 24 h after stimulation in any of the analyzed cells, including IL-2-independent ED-40515(−), ED-70423(−), ED-50823(−), ATL-55 T(−), and ATL-72/2(−) cells^22^. These findings suggest that MT-50.4 cells were derived from CD13^+^ HTLV-1-infected T cells in vivo. Notably, both cell lines expressed Foxp3, although the expression level was higher in MT-50.4 than in MT-50.1 cells. Most HTLV-1-infected cell lines reported thus far were established using whole peripheral T cells, including both Tregs and non-Tregs. However, most of those cell lines showed neither Foxp3 expression nor immunosuppressive functions^12^. HTLV-infected cell lines that sustain high Foxp3 expression may be derived from Tregs^12,29,30^. Therefore, we considered that MT-50.1 and MT-50.4 cells originated from CD13^−^ HTLV-1-infected Tregs and CD13^+^ HTLV-1-infected Tregs, respectively. Furthermore, the CD13^+^Foxp3^+^ Tregs subpopulation with enhanced inhibitory activity has been identified in the human peripheral blood^16^. Thus, CD13^−^ MT-50.1 and CD13^+^ MT-50.4 cells represent unique HTLV-1-infected cell lines with phenotypes similar to those of Tregs.

The major strength of the present study was that the paired availability of the syngeneic models enabled the examination of the functional role of CD13 expression in HTLV-1-infected cells with Treg-like phenotypes. In contrast to MT-50.1 cells, MT-50.4 cells produced high levels of IL-10 and TGF-β. These immunosuppressive cytokines are key mediators of Treg function, and peripherally-induced Tregs appear to modulate peripheral immune tolerance by expressing IL-10 and TGF-β^9,31^. Furthermore, we demonstrated that MT-50.4 cells, similar to PBMC-derived Tregs, inhibited the proliferation of stimulated CD4^+^CD25^−^ T cells significantly more than MT-50.1 cells. These findings suggest that CD13^+^Foxp3^+^CD4^+^CD25^+^ HTLV-1-infected cells represent a subpopulation with increased immunosuppressive function. Whether such cells lead to a more profound immunosuppressive environment enabling them to escape from the host immune response needs to be investigated further.

Another notable finding in the present study was that bestatin (or ubenimex), which is a potent CD13 inhibitor, induced a significant attenuation of MT-50.4 cell growth through the induction of G0/G1 cell cycle arrest, while it did not for MT-50.1 cells. These findings suggest that targeted inhibition of CD13 is likely beneficial for antiproliferative activity in cells with high CD13 expression levels. Reducing the Treg number is expected to enhance the efficacy of antitumor therapies^32,33^. In addition, inhibition of CD13 decreases the activity of CD13^+^ Tregs, which is associated with tumor progression^16^. Thus, there is a growing clinical need to selectively target Tregs in tumors. Regarding ATL, although the frequency of Foxp3^+^ cells differs among cases^14^, our present findings would promote studies on the efficacy of bestatin for reducing CD13^+^Foxp3^+^ Tregs.

Our study had some limitations. The generalization of the present study results may be limited because the findings were obtained using the two cell lines established in the study. To address this limitation, a systematic investigation of a large number of patients with ATL and individuals with non-malignant HTLV-1 infection is required to determine the frequency of CD13 expression. In addition, the isolation of HTLV-1-infected CD13^+^Foxp3^+^ cells from the peripheral blood needs to be performed to investigate their immunosuppressive functions further. However, the paucity of naturally occurring Tregs may hamper the studies of the specific type of Tregs. Furthermore, the studies of Tregs were also limited due to the scarcity of reported HTLV-1-infected Treg cell lines. Although some differences may exist in the phenotype and function between MT-50.4 cells and primary HTLV-1-infected CD13^+^ Tregs, the MT-50.4 cell line is valuable for studies of CD13^+^ Tregs, which may have a role in immune suppression in ATL. Second, the exact reason for the CD13 negative status of the primary ATL cells in our patient remains unclear. HTLV-1-infected CD13^−^ clone may have acquired malignant phenotypes leading to the development of ATL tumor. Furthermore, Karube et al.^5,6^ suggested that Foxp3 expression may be lost during disease progression or HTLV-1-induced malignant transformation. Conversely, the non-leukemic MT-50.4 cells could retain the CD13^+^Foxp3^+^ phenotype. Finally, we could not fully exclude the possibility that HTLV-1 infection in vivo or during cell culture induced aberrant CD13 expression in MT-50.4 cells, while we established the CD13-negative MT-50.1 cell line with the same conditions as the MT-50.4 cell line. Despite these limitations, our findings are expected to stimulate further studies to investigate the effect of CD13 expression in HTLV-1-infected Foxp3^+^ cells on enhanced immunosuppression function.

In summary, we present a novel HTLV-1-infected CD13^+^Foxp3^+^ cell line, MT-50.4, exhibiting an increased Treg-like activity, including the production of high levels of immunosuppressive cytokines and suppression of conventional CD4^+^ T cells. The cell line will allow more in-depth studies of HTLV-1-infected CD13^+^Foxp3^+^ cells and provide a model for discovering therapeutic candidates targeting CD13 for controlling CD13^+^ Treg numbers.

### Supplementary Information

Supplementary Information.

## Data Availability

The data that support the findings of this study are available from the corresponding author upon reasonable request.
