# Supplementary material for: Novel paired CD13-negative (MT-50.1) and CD13-positive (MT-50.4) HTLV-1-infected T-cell lines with differential regulatory T cell-like activity
Source: Sci Rep. 2024 May 31;14:12549. doi: 10.1038/s41598-024-63494-x (PMC11143202; doi:10.1038/s41598-024-63494-x)
Supplement: Supplementary file 1 — Supplementary Information. [file 41598_2024_63494_MOESM1_ESM.pdf]

Supplemental Information

Novel paired CD13-negative (MT-50.1) and CD13-positive (MT-50.4) HTLV-1-infected T-cell lines with differential regulatory T cell-like activity

Yuki Egawa[1], Tomonori Higuchi[1], Yumiko Hashida[1], Kazuyuki Ueno[1], Kensuke Kojima[2], Masanori Daibata[1,*]

[1]Department of Microbiology and Infection, Kochi Medical School, Kochi University, Nankoku, Kochi 783-8505, Japan.

[2]Department of Hematology, Kochi Medical School, Kochi University, Nankoku, Kochi 783-8505, Japan.[3]Science Research Center, Kochi University, Nankoku, Kochi, Japan

*Corresponding author

Email: daibatam@kochi-u.ac.jp

Supplementary Table S1. Mouse monoclonal antibodies used for immunohistochemistry.

Antibody name	Clone ID	Source
Anti-human CD3	SP7	Nichirei Biosciences
Anti-human CD4	1F6	Leica Biosystem
Anti-human CD8	4B11	Leica Biosystem
Anti-human CD13	2D8D11	Proteintech
Anti-human CD20	L26	Nichirei Biosciences
Anti-human CD25	4C9	Nichirei Biosciences

Supplementary Table S2. Sequences of primers used for qRT-PCR.

Target region	Primer name	Sequence (5′→3′)	Nucleotide position	Accession number
tax	Tax-F	CCAACACCATGGCCCACTT	4821−7123	NC_001436.1
	Tax-R	GATGGGGTCCCAGGTGATCT		
HBZ	HBZ-F	ATGGCGGCCTCAGGGCTGTT	8328–6765	NC_001436.1
	HBZ-R	GCGGCTTTCCTCTTCTAAGG		
ACTB	ACTB-F	GTGGATCAGCAAGCAGGAGTATGA	1257–1342	NM_001101.4
	ACTB-R	TAGGTTTTGTCAAGAAAGGGTGTAA		

Supplementary Table S3. Monoclonal antibodies used for flow cytometry.

Antibody name (clone ID)	Fluorescent-labeled	Source
Anti-human CD4 (RPA-T4)	FITC	BioLegend
Anti-human CD13 (WM15)	PerCP-Cy5.5	BioLegend
Anti-human CD25 (BC96)	APC	BioLegend
Anti-human CD127 (A019D5)	APC/Fire 750	BioLegend
Anti-human FOXP3 (PCH101)	PE	Thermo Fisher Scientific
Isotype-matched mouse IgG_1 (MOPC-21)	PerCP-Cy5.5	BioLegend
Isotype-matched rat IgG_{2a} (eBR2a)	PE	Thermo Fisher Scientific

Abbreviations: APC, allophycocyanin; FITC, fluorescein isothiocyanate; PE, Phycoerythrin; PerCP-Cy5.5, peridinin chlorophyll protein-cyanine 5.5.

Supplementary Table S4. Monoclonal antibodies used in Supplementary Figure S4.

Antibody name (clone ID)	Fluorescent-labeled	Source
Anti-human CD2 (S5.2)	FITC	BD Bioscience
Anti-human CD3 (SK7)	PE	BD Bioscience
Anti-human CD4 (SK3)	APC-H7	BD Bioscience
Anti-human CD5 (L17F12)	FITC	BD Bioscience
Anti-human CD7 (M-T701)	APC	BD Bioscience
Anti-human CD8 (SK1)	BV510	BD Bioscience
Anti-human CD10 (HI10a)	PE	BD Bioscience
Anti-human CD11c (B-ly6)	BV510	BD Bioscience
Anti-human CD13 (L138)	PE	BD Bioscience
Anti-human CD14 (MφP9)	APC-H7	BD Bioscience
Anti-human CD16 (3G8)	BV510	BD Bioscience
Anti-human CD19 (HIB19)	BV421	BD Bioscience
Anti-human CD20 (L27)	APC-H7	BD Bioscience
Anti-human CD22 (HIB22)	BV421	BD Bioscience
Anti-human CD23 (M-L233)	BV421	BD Bioscience
Anti-human CD25 (M-A251)	BV421	BD Bioscience
Anti-human CD30 (Ber-H83)	FITC	BD Bioscience
Anti-human CD34 (8G12)	APC	BD Bioscience
Anti-human CD38 (HB7)	APC-H7	BD Bioscience
Anti-human CD41 (HIP8)	BV510	BD Bioscience
Anti-human CD56 (NCAM16.2)	APC	BD Bioscience
Anti-human CD79a (HM47)	BV42	BD Bioscience
Anti-human CD103 (Ber-ACT8)	BV510	BD Bioscience
Anti-human MPO (MPO-7)	PE	Agilent Technologies
Isotype-matched mouse IgG_1 (X40)	FITC, PE	BD Bioscience

Abbreviations: APC, allophycocyanin; BV, Brilliant violet; FITC, fluorescein isothiocyanate; PE, Phycoerythrin.

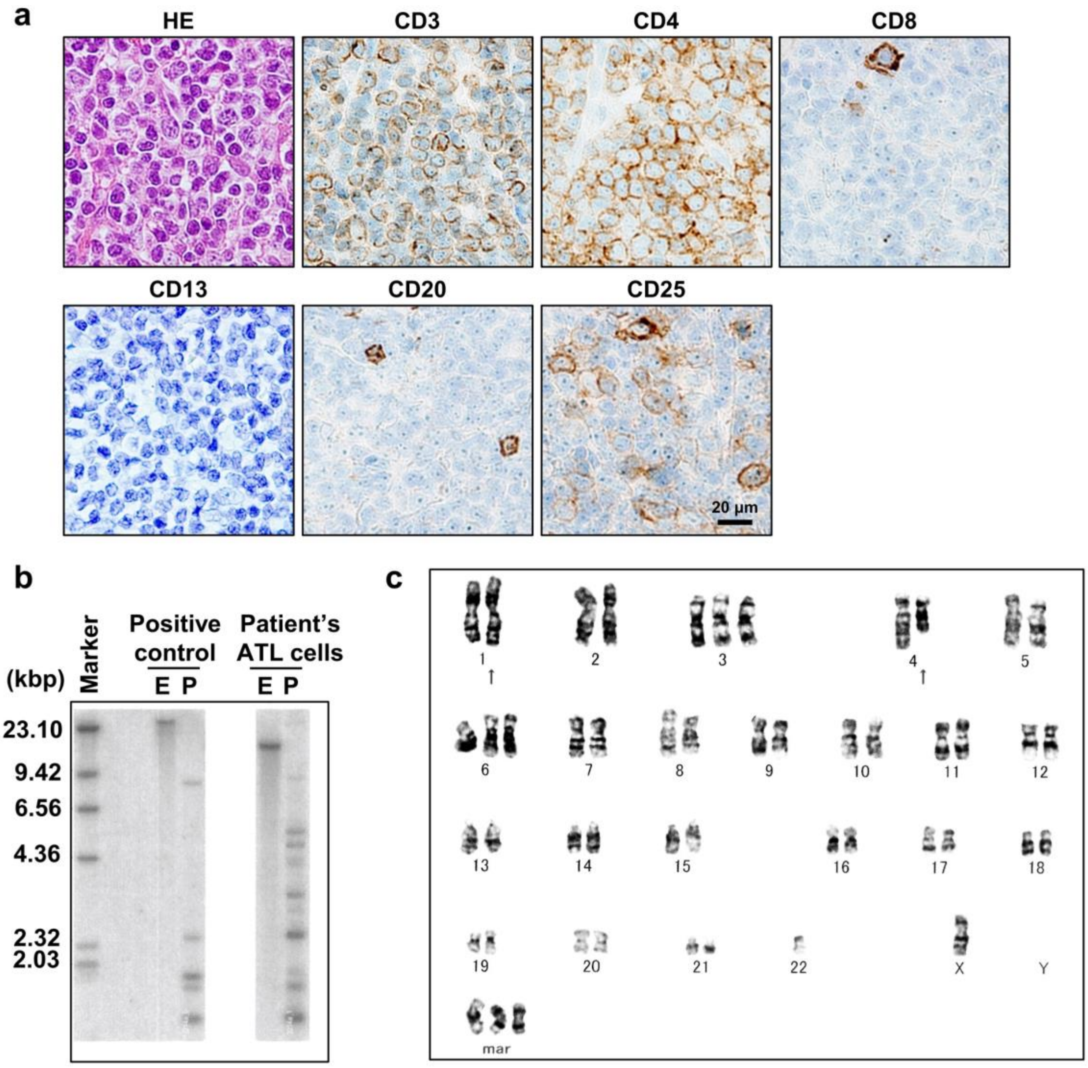

Supplementary Figure S1. Characteristics of the patient's ATL cells. (**a**) Hematoxylin and eosin (HE) staining of ATL cells in the affected lymph node. Immunohistochemistry showed positive staining for CD3, CD4, and CD25 and negative staining for CD8, CD13, and CD20 in the tumor cells. (**b**) HTLV-1 clonality assay in the patient's ATL cells, showing monoclonal integration of the HTLV-1 provirus genome. High molecular weight DNA was digested using *Eco*RI (E) or *Pst*I (P). The digests were separated using agarose gel electrophoresis, blotted onto a nitrocellulose filter, and hybridized with an HTLV-1 probe. This analysis was conducted in the clinical laboratory when the patient was admitted to our hospital and the blot photograph was obtained from the patient's medical record. (**c**) Giemsa-banded karyotype, showing the following karyotype: 49, X, −X, add(1)(q32), +3, del(4), +6, −22, +3mar. Arrows indicate structural chromosomal abnormalities.

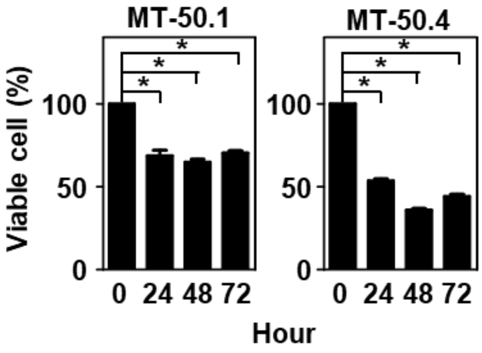

Supplemental Figure S2. IL-2-dependent proliferation of MT-50.1 and MT-50.4 cells. Cells were cultured without IL-2 for 72 h, and viable cells were counted every 24 h using a flow cytometer by gating out cells stained with propidium iodide. The graphs show the percentage of viable cells in the total cell population. All experiments were independently repeated more than three times. Data are shown as the mean ± SEM. Significant differences are shown as $^*P < 0.05$.

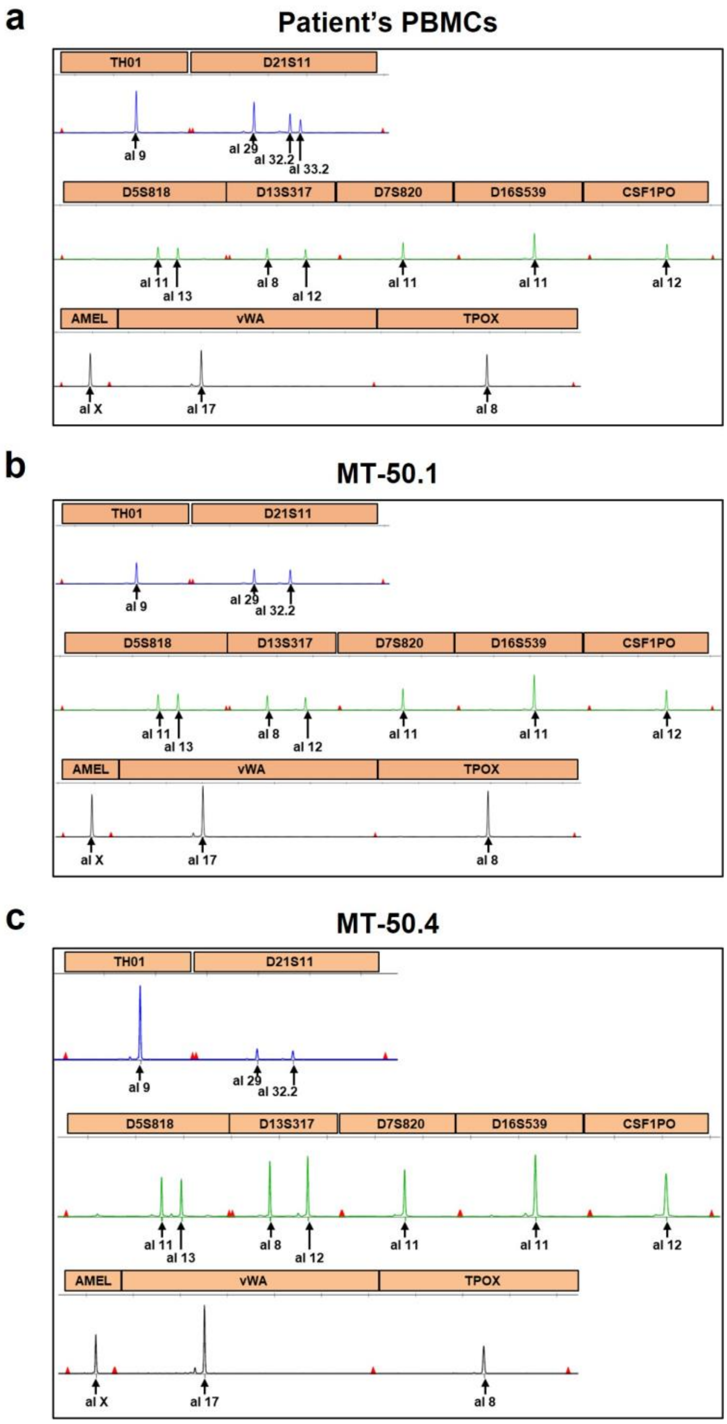

Supplementary Figure S3. Confirmation of the authenticity of MT-50.1 and MT-50.4 cells using short tandem repeat (STR) DNA fingerprinting. Ten STR loci were selected to compare the patient's peripheral blood mononuclear cells (PBMCs) and the established cell lines. These loci collectively provided a genetic profile with a random match probability of 1 in 2.92×10^9, according to the manufacturer's instructions (https://www.future-science.com/doi/10.2144/000114014). (**a**) Patient's PBMCs. (**b**) MT-50.1 cells. (**c**) MT-50.4 cells.

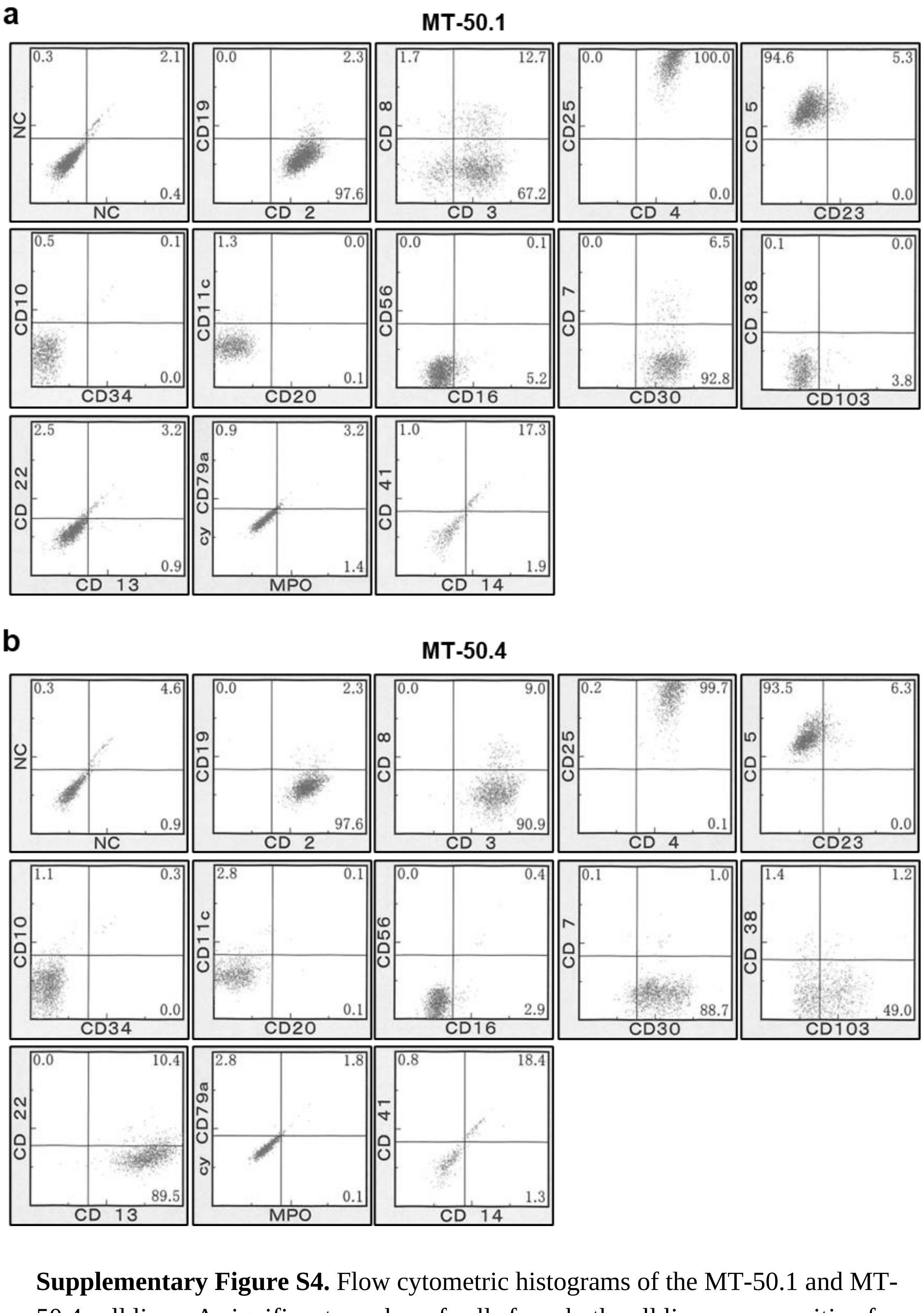

Supplementary Figure S4. Flow cytometric histograms of the MT-50.1 and MT-50.4 cell lines. A significant number of cells from both cell lines were positive for CD2, CD3, CD4, CD5, CD25, and CD30, while only MT-50.4 cells strongly expressed CD13. NC: negative control, mouse IgG. (**a**) MT-50.1 cells. (**b**) MT-50.4 cells.

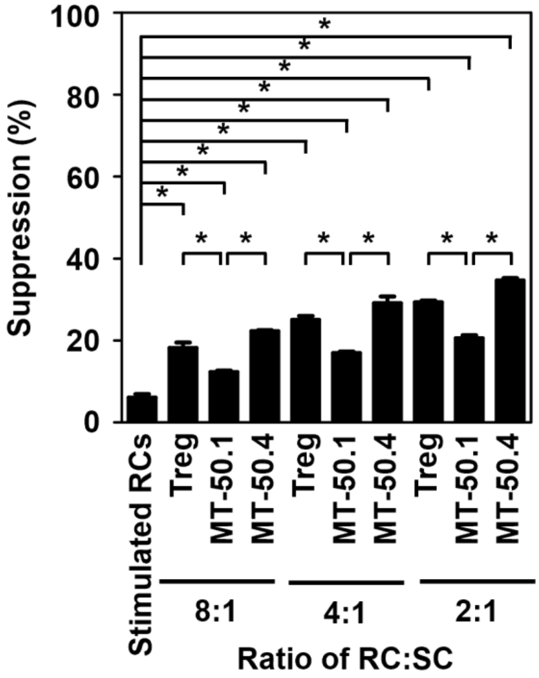

Supplementary Figure S5. Suppressive activity of MT-50.1 and MT-50.4 cells. $CD4^{+}CD25^{-}$ T cells (5×10^{4}, responder cells [RCs]) were isolated from PBMCs of a healthy donor (donor 2). Carboxyfluorescein succinimidyl ester (CFSE)-labeled stimulated $CD4^{+}CD25^{-}$ T cells were cocultured with suppressor cells (SCs; autologous PBMC-derived Treg cells, MT-50.1, or MT-50.4 cells) at RC:SC ratios of 8:1, 4:1, and 2:1. After 5 days of culture, the CFSE intensity of RCs was measured using a flow cytometer. The bar graph shows the percent suppression of cell division. The suppression levels induced by MT-50.4 cells were significantly higher than those by MT-50.1 cells. Data are shown as the mean ± SEM of three independent experiments. Significant differences are shown as $*P < 0.05$.

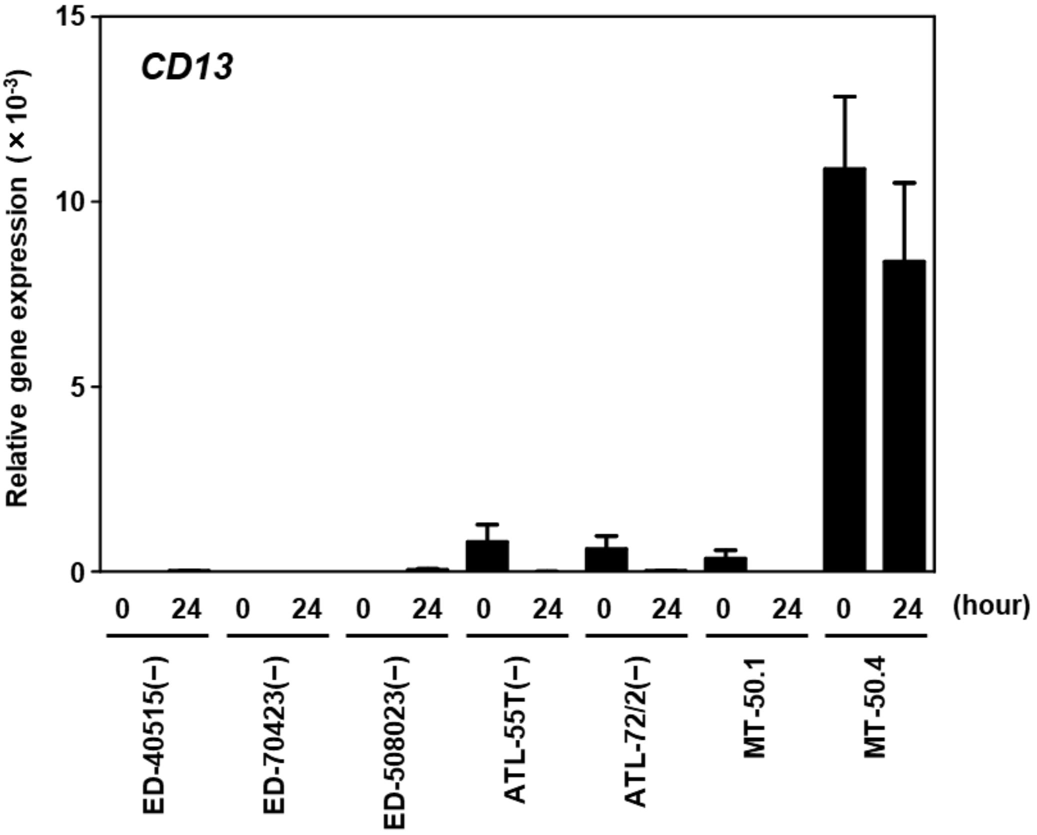

Supplementary Figure S6. IL-2 stimulation for possible *CD13* mRNA expression in CD13⁻ HTLV-1-infected cells. Cells were incubated with 100 U/mL of IL-2 (Shionogi Pharma, Osaka, Japan) for 24 h at 37 °C with 5% CO_2 in the air. Total RNA was extracted and purified using TRIzol reagent (Thermo Fisher Scientific, Tokyo, Japan) and Direct-zol DNA/RNA miniprep kits (Zymo research, Irvine, CA, USA). The total RNA was treated with DNase to avoid any amplification of genomic DNA and was reverse-transcribed using SuperScript IV VILO master mix (Thermo Fisher Scientific). The reaction was conducted on a StepOnePlus thermocycler (Thermo Fisher Scientific) with KOD SYBR qPCR mix (Toyobo, Osaka, Japan) containing 0.4 μM of each primer. The primer sequences used to determine the *CD13* expression are as follows: 5′-TGCAGCAAAGAGTTGTGGATCC-3′ (forward) and 5′-ACCTTGCCCAATGACGTTGTTG-3′ (reverse). The PCR conditions were 2 min at 98 °C, followed by 45 cycles of 10 s at 98 °C, 10 s at 62 °C, and 30 s at 68 °C. Relative gene expression levels were calculated using $2^{-\Delta Ct}$ values, with the β-actin gene (*ACTB*) as a housekeeping control. The five IL-2-independent CD13⁻ HTLV-1-infected cells were used in this study: ED-40515(–), ED-70423(–), ED-50823(–), ATL-55T(–), and ATL-72/2(–).

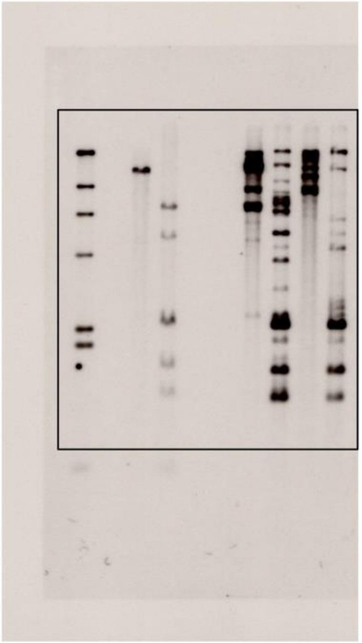

Supplementary Figure S7. The original photograph of Figure 2a showing the full-length membrane, with the membrane edges visible. Crop lines are indicated in the photo.

Supplementary Methods

Immunohistochemistry. Immunohistochemistry was performed on formalin-fixed paraffin wax-embedded (FFPE) biopsied tissue sections. FFPE sections were dewaxed and rehydrated, and antigen retrieval was performed by heating at 95°C for 30 min in Target Retrieval Solution, pH 6 (Agilent Technologies, Tokyo, Japan). The sections cooled to room temperature were treated with methanol containing 0.5% hydrogen peroxidase for 20 min to inactivate endogenous peroxidase. The sections were incubated with Histofine 10% normal goat serum (Nichirei Biosciences, Tokyo, Japan) for 20 min to block nonspecific reactions, and treated overnight at 4°C with each mouse monoclonal antibody listed in Supplementary Table S1. The sections were incubated with Histofine simple stain MAX PO (Nichirei Biosciences), and signals were detected using the Liquid DAB+ Substrate Chromogen System (Agilent Technologies). The nuclei were counterstained with hematoxylin.
